# Functional luminal imaging probe: a new technique for dynamic evaluation of mechanical properties of the anal canal

**DOI:** 10.1007/s10151-012-0871-0

**Published:** 2012-08-31

**Authors:** F. Luft, L. Fynne, H. Gregersen, F. Lundager, S. Buntzen, L. Lundby, S. Laurberg, K. Krogh

**Affiliations:** 1 Neurogastroenterology Unit, Department of Hepatology and Gastroenterology V, Aarhus University Hospital, Norrebrogade, 8000 Aarhus, Denmark; 2 Department of Surgery P, Aarhus University Hospital, Aarhus, Denmark; 3 Sino-Danish Center for Education and Research, Aarhus, Denmark; 4 Sino-Danish Center for Education and Research, Beijing, China; 5 Mech-Sense, Department of Gastroenterology, Aalborg Hospital, Aalborg, Denmark

**Keywords:** Anal canal, Biomechanical properties, Functional luminal imaging probe, Fecal incontinence

## Abstract

**Background:**

The muscle structures surrounding the anal canal are of major importance in maintaining continence but their anatomy and function vary along its length. Standard manometry does not provide detailed information about mechanical properties of the anal canal. A new functional luminal imaging probe (FLIP) has been developed for this purpose. The aim of our study was to investigate whether FLIP allows detailed evaluation of dynamic biomechanical properties along the length of the anal canal.

**Methods:**

The in vitro validity and reproducibility of the FLIP system were tested. Fifteen healthy volunteers (age 32–65 years, mean 51 years), of whom 12 were females, were investigated. The integrity and dimensions of the anal sphincter apparatus were evaluated with endoanal ultrasonography and standard anal manometry. During standardized distensions with the FLIP, 16 cross-sectional areas of the anal canal were measured at 5-mm intervals. Distensibility of the following three segments was evaluated: upper anal canal (surrounded by the internal anal sphincter and the puborectalis muscle), mid-anal canal (surrounded by the internal anal sphincter and the external anal sphincter) and lower anal canal (surrounded by the external anal sphincter). Color contour plots were generated from the FLIP-based dynamic recordings of serial cross-sections.

**Results:**

In vitro tests confirmed the validity and reproducibility of the FLIP system. The luminal geometry during distension and the biomechanical properties of the anal canal differed at the three levels. Both at rest and during squeeze the mid-anal canal was significantly less distensible than the upper (*p* < 0.01) and the lower (*p* < 0.05) anal canal.

**Conclusions:**

FLIP is a promising method for evaluation of the nonhomogeneous biomechanical properties along the length of the anal canal.

## Introduction

Fecal incontinence is a debilitating condition affecting approximately 2 % of the general population [1]. Maintaining anal continence requires complex interactions between the muscle complex surrounding the anal canal, anorectal sensibility, colorectal motility, stool consistency and rectal wall properties. The anal canal is approximately 3.5 cm long, and the surrounding structures are inhomogeneous [2]. The upper part of the anal canal is surrounded by the smooth muscle internal anal sphincter (IAS) and the striated puborectalis muscle. The middle part is surrounded by the IAS but also the striated external anal sphincter (EAS) muscle and the very distal part is surrounded by the EAS only [3]. The main function of the IAS is to generate 50–85 % of the anal resting pressure and to relax at defecation [4, 5]. The main function of the EAS is voluntary contraction to avoid inappropriate flatus or defecation [4–6]. The anatomical differences along the sphincter complex indicate that the biomechanical properties vary along the anal canal.

Several methods exist for evaluation of anal sphincter morphology and physiology. Magnetic resonance imaging (MRI) and transanal ultrasonography are commonly used for description of the morphology [7]. Anal manometry is generally used to measure the length of the high pressure zone in the anal canal and to determine the anal resting and squeeze pressures [4, 6]. In general, the anal resting pressure is taken as a measure of IAS function while the pressure increment at squeeze reflects the function of the EAS. Anal manometry is easy to perform but the method is hampered by large intersubjective variation and overlap between healthy subjects and various patient groups [8]. Existing techniques for evaluation of anal muscle structures and physiology mainly assess static properties. This may be inexpedient since both fecal incontinence and defecation are dynamic events and anal sphincter muscles have dynamic properties [5]. Furthermore, the heterogeneous structures of the muscle complex surrounding the anal canal call for evaluation of the biomechanical properties at different locations in the anal canal.

The recently developed Functional luminal imaging probe (FLIP) for assessing sphincter mechanics in the gastrointestinal tract allows determination of serial cross-sectional areas (CSAs) during distension. This provides detailed and segmental description of geometric and mechanical properties. FLIP was originally designed to study dynamic wall properties at the gastro-esophageal junction especially in patients with achalasia [9–11]. Our hypothesis for the present study was that dynamic properties at distension vary at different locations in the anal canal. Therefore, the aim of the present paper was to use FLIP for evaluation of segmental mechanical properties of the anal canal in healthy volunteers.

## Materials and methods

After informed consent, 15 healthy subjects (12 female) aged 32–65 years (mean 51 years) were studied with FLIP, endoluminal anal ultrasonography and standard anal manometry. None of the subjects had any history of perianal disease or surgery, and all had normal bowel function. Female participants had given birth 0–3 times but had no history of obstetric sphincter lesions or instrumentation during delivery. The study was conducted according to the Helsinki II declaration and approved by the Local Ethics Committee (M-20100217).

### Functional lumen imaging probe (FLIP)

**Fig. 1:**
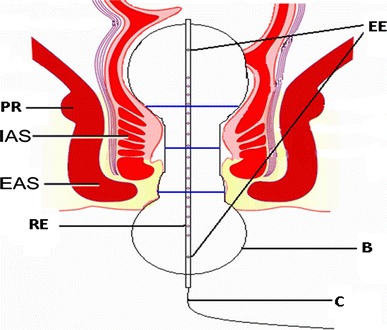
Schematic representation of FLIP within the anal canal. *PR* puborectalis, *IAS* internal anal sphincter, *EAS* external anal sphincter, *EE* excitation electrodes, *RE* recording electrodes, *B* bag, *C* catheter. The *blue lines* indicate the upper (at the level of the puborectalis muscle), mid- and lower (at the level of the most distal part of the external sphincter) anal canal

Functional lumen imaging probe is based on the impedance planimetry principle [12–14]. By impedance planimetry, the luminal CSA of an organ is determined from the electrical impedance between a pair of detection electrodes placed in a balloon or bag infused with electrically conductive fluid. By inflating the bag and determining corresponding CSAs, localized biomechanical properties can be evaluated. EndoFLIP^®^ Imaging System (Crospon Inc., Galway, Ireland) for investigation of the gastrointestinal sphincteric regions consists of a two lumen polyethylene catheter with an outer diameter of 3 mm (Fig. 1). It determines and records the CSA (diameter) at 16 serial locations 5 mm apart, thereby covering a total length of 8 cm. A 12-cm long noncompliant cylindrical bag is mounted on the probe and folded at the ends before fixation to avoid displacement. Therefore, the catheter can move up to 3 cm within the bag without affecting the balloon shape itself. The bag is filled with a saline solution at a ramp inflation rate of 40 ml/min to a maximum inflation volume of 50 ml. The bag pressure is determined by a solid state pressure transducer placed on the probe inside the bag. Data are recorded at a sampling rate of 10 Hz and transferred offline to a personal computer where it is displayed by a custom made data acquisition software programmed in Labview (National Instruments, Austin, TX, USA). The diameter range that can be measured with the probe is 4.3–35.0 mm.

In vitro validation of the system was performed before the in vivo studies to ensure accuracy and reproducibility of the measurements. For this purpose, FLIP was inserted and inflated inside 5 straight tubes and 2 tubes with abruptly changing diameter. During each insertion, the bag was inflated 10 times, and diameters measured were recorded for 30 s. This procedure was performed on 7 consecutive days and again on day 15.

During in vivo studies digital rectal examination before investigation ensured that the rectal ampulla was empty. The subjects were placed in the left lateral position, and the probe was gently inserted into the anal canal in such a way that two detection electrodes were visible outside the anal verge (Fig. 1). During the experiments, the probe was held in place by one of the investigators. The bag was first inflated two times with 10 ml to ensure proper unfolding of the bag. Afterward, it was inflated with 20, 30, 40, and 50 ml. The bag was emptied between distensions. At the end of each distension step, the subjects were asked to squeeze for a short period of 10 s. The position of FLIP was checked visually by the investigator after each squeeze. In case of dislocation of the probe, the displacement (number of electrodes visible outside the anal verge) was written down, and the probe was repositioned after deflation.

In a pilot study, the positioning of the electrode array within the bag was checked with transcutaneous ultrasonography in a single subject. It was verified that the electrode array stayed in the center of the bag, parallel to the walls of the anal canal. Further, it was verified that distension of the distal rectum to the maximum possible diameter reached with FLIP (35 mm) only caused a very minor relaxation of the anal canal lasting a few seconds.

### Endoanal ultrasonography

Endoanal ultrasonography was performed with the FlexFocus (BK Medicals, Harlev, Denmark). Three-dimensional reconstructions were made, and two experienced consultant colorectal surgeons individually evaluated the integrity of the sphincter apparatus.

### Anal manometry

Standard stationary pull through anal manometry (Duet, Mediwatch PLC, Rugby, UK) was performed on all subjects prior to FLIP. Anal resting pressure, anal squeeze pressure and anal squeeze pressure increment were recorded. Technical details and normal values for anal manometry at our laboratory have previously been published [6].

### Data analysis

The in vitro data were analyzed using the deviation of measured diameter from the real value in percent as a measure of the accuracy of the system and the day-to-day coefficient of variance as a measure for reproducibility.

For analysis and reconstruction of the three-dimensional shape obtained by FLIP, custom-made analysis software was programmed in MATLAB R2009b (Mathworks, Natick, MA, USA). The distance of 5 mm between electrode pairs did not allow precise estimation of the length of the anal canal. Therefore, a mathematical function of the shape was made through interpolation with an accuracy of 0.1 mm. After determining the smallest diameter of the anal canal, the algorithm then determined the level both proximally and distally where the diameter increased more than a set value in percentage of the narrowest diameter. As the shape of the anal canal changed during distension, the set value had to be adjusted accordingly. The set value for increase in diameter with reference to the narrowest point was 60 % at 20 and 30 ml, 40 % at 40 ml and 20 % and 50 ml. From the length determined by the algorithm, the anal canal was divided into a lower, middle, and upper part. At each of the three locations, the lowest diameter was measured, and wall tension was computed at the end of each inflation volume (20, 30, 40 and 50 ml). Wall tension (*T*) was calculated according to Laplace’s law $T = P \cdot r$, where *P* is the distension pressure recorded with FLIP and *r* the measured radius.

### Statistical analysis

The diameters measured at the mid-anal canal were compared to the diameters measured at the upper and lower anal canal using a paired-*t* test. This was done for the diameters measured during rest and during squeeze. Furthermore, a paired-*t* test was performed to compare the diameters measured at the upper and lower anal canal. All analysis was done using Matlab (Mathworks, Natick, MA, USA). A *p* value of ≤0.05 was considered statistically significant.

## Results

### In vitro tests

The diameter of the bag was measured in straight tubes with a numerical deviation from the true value of 4.4 % (range −4.3 to 4.7 %) and in tubes with a narrowing section to simulate a sphincter region with 7.4 % (range −4.5 to 10.3 %). The error in percent was largest for the smallest tube, reflecting a deviation from the true value of less than 1 mm. Furthermore, the day-to-day variance over a period of 15 days was 3.3 % (range 1.2–5.3 %).

### Endoanal ultrasonography and anal manometry

Endoanal ultrasonography revealed no sphincter defects or other pathology. Anal resting pressure was 32–133 cm H_2_O (median 67 cm H_2_O), anal squeeze pressure was 82–252 cm H_2_O (median 145 cm H_2_O), and pressure increment during squeeze was 25–200 cm H_2_O (median 92 cm H_2_O).

### Configuration of the anal canal at distension

**Fig. 2:**
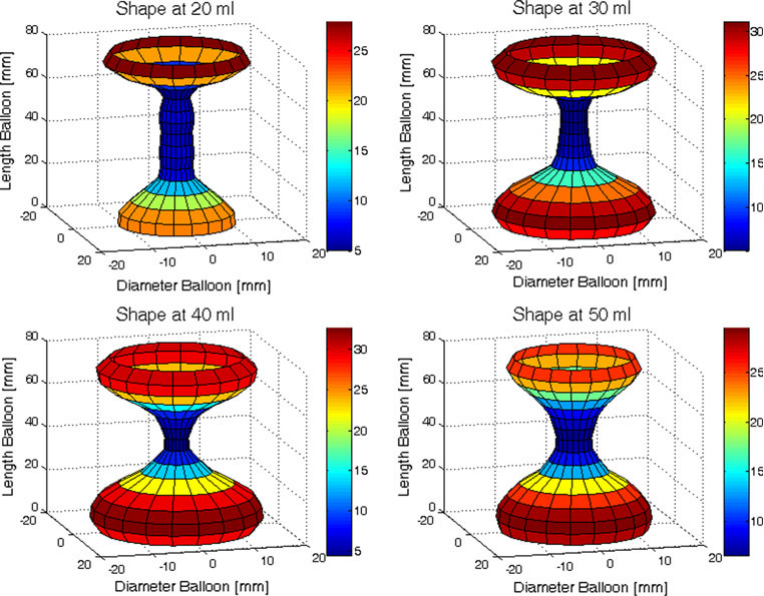
The anal canal visualized with FLIP (*EP* electrode pair). Three-dimensional shape of the anal canal at rest during distension with 20 (*top left*), 30 (*top right*), 40 (*bottom left*) and 50 (*bottom right*) ml. The *colors* indicate the diameter measured at a specific position. In the upper and lower parts where the diameters are large (approximately 20–24 mm) the balloon is in the rectum or outside the anal verge. The closed part of the anal canal itself (*blue area*) remains at a minimum diameter (around 5 mm) during distension with 20, 30 and 40 ml, but increases in diameter during distension with 50 ml. At 20 ml the small impression made by the puborectalis muscle is seen at the upper end of the anal canal

**Fig. 3:**
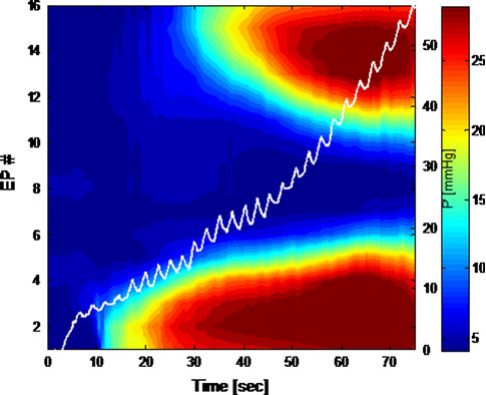
Color contour plot of the anal canal during a single inflation up to 50 ml. The *white line* represents the pressure within the bag (mmHg). The *colors* indicate the diameter present at a specific point in time at a specific electrode pair (EP). The *red/brown colors* show the large diameters of the bag in the rectum (*top*) and outside the anal verge (*bottom*). The first 30 s (*x*-axis) represents filling of the bag without distension of the anal canal. After that the *dark blue color* represents the closed part of the anal canal. After 30 s a change in diameter at the upper part is noticed (from *dark blue* to a *lighter blue*). The same *curve* is seen in the lower anal canal but with less steep a slope. The fully closed part of the anal canal (*dark blue*) shortens and after 70 s the mid-anal canal (at EP 8) is the only part to remain closed (*dark blue*)

Three-dimensional (3D) reconstructions at distension volumes of 20, 30, 40 and 50 ml from a single subject at rest are shown in Fig. 2. At rest, the anal canal opened during inflation but the distension pattern was not uniform. In all subjects, the upper anal canal opened first. This was followed by opening of the lower anal canal while the mid-anal canal had the greatest resistance usually only yielding at 50 ml. The color contour plot shown in Fig. 3 includes the dynamic information from a single distension illustrating the same distension pattern that the most distensible part of the anal canal was the upper part, while the mid-part was the least distensible.

### Biomechanical properties of the anal canal

**Fig. 4:**
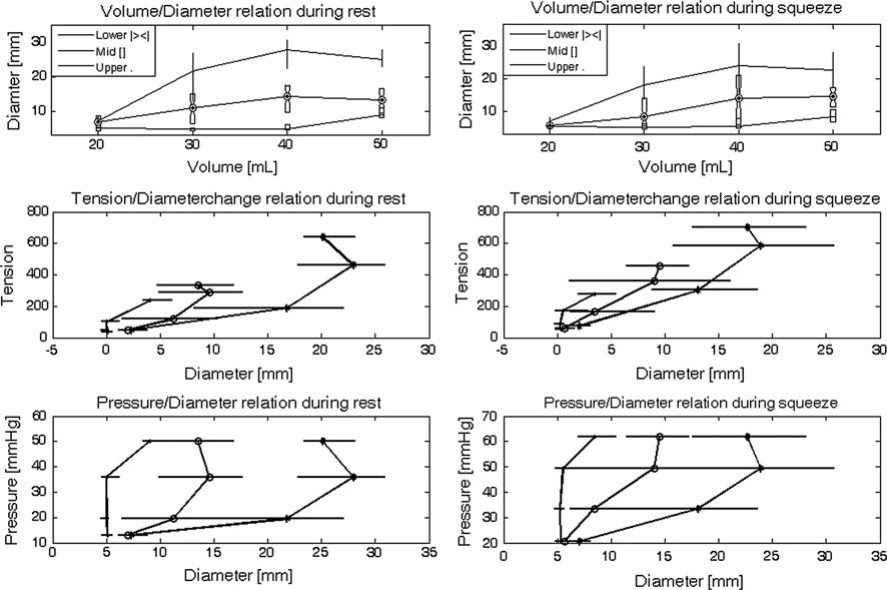
Diameter/volume relationship during rest (*top left*) and squeeze (*top right*). Tension/diameter relationship during rest (*middle left*) and squeeze (*middle right*) and pressure/diameter relationship during rest (*bottom left*) and squeeze (*bottom right*) [lower (*open circle*), mid-(*dot*) and upper (*asterisk*) anal canal]. Data shown are as median with 25 and 75 % quartiles

Geometric and biomechanical properties of the upper, mid- and lower anal canal are shown in Fig. 4. The properties were analyzed by calculation of the diameter/volume, diameter change/tension and diameter/pressure relationships during rest and squeeze. At rest, the diameters measured during distension of the mid-anal canal were significantly smaller than the diameters measured in the lower (*p* < 0.05) and upper (*p* < 0.01) anal canal. Furthermore, the diameters measured at the lower anal canal were significantly smaller than those of the upper anal canal (*p* < 0.04), except for distension at 20 ml.

During squeeze, the mid-anal canal was also significantly less distensible than the lower (*p* < 0.05) and upper anal canal (*p* < 0.01). Also during squeeze, the diameters measured at the lower anal canal were significantly smaller compared with the diameter measured at the upper anal canal (*p* < 0.05) except for a distension volume of 20 ml (*p* = 0.06). No statistically significant difference in diameter was determined by comparing the resting and squeeze diameters with each other, except for the lower anal canal at a distension volume of 20 ml (*p* = 0.01). In all cases except one, the pressures measured in the bag during squeeze were not higher than the maximum squeeze pressure determined with anal manometry.

## Discussion

The most important result from the present study is that the geometry of the lumen and the biomechanical properties of the anal canal are not uniform during distension. This is in accordance with the variation in anatomical structures surrounding the anal canal [3] and with data obtained using anal manometry. At rest, the least distensible part was clearly the mid-anal canal which is surrounded by both the IAS and EAS muscles. Our results strongly indicate that the mid-anal canal is the most important part in maintaining continence at rest. The upper part of the anal canal was the most compliant. It is fully surrounded by the IAS while the puborectalis muscle forms a sling supporting the posterior half of the circumference. During squeeze, the diameters of the anal canal changed but differences did not reach statistical significance. Like at rest, biomechanical properties during squeeze were not uniform with the mid-anal canal being the least distensible.

The FLIP procedure was easy to perform and well tolerated by all subjects. The standard method for evaluation of anal sphincter function is manometry and a number of techniques and protocols for this exist [1]. Manometry is also easy to perform, but results have to be interpreted with care, as intersubjective variation is very high, and there is poor correlation between anal pressures and the clinical state of subjects investigated [15]. Manometry and FLIP evaluate different aspects of anal sphincter function. The major difference is that FLIP determines the resistance to distension of the anal canal. It remains to be shown whether this parameter adds clinically important information to pressure obtained by standard manometry. Thus, the present study does not propose FLIP as an alternative to manometry. Rather, we present a method for obtaining additional information about anal physiology.

The FLIP is based on the impedance planimetry principle where luminal cross-sectional area is computed from the impedance within a fluid-filled bag. Previous studies of rectal compliance have shown that elongation of a balloon in the rectum may cause significant overestimation of rectal compliance and that this source of error is avoided with impedance planimetry [16]. There are, however, other substantial sources of error with impedance planimetry. Thus, the probe should be in parallel with the wall and located centrally in the lumen. These sources of error are significant in large diameter organs including the rectum but their effect in smaller organs like the anal canal is minimal.

We find that methods for investigation of the anal canal function should reflect the nonuniform mechanical properties. Previously, Rasmussen et al. [8, 17] published a method of performing dynamic anal manometry. Even though the method provided more detailed information than manometry, it has not been extensively used. In our opinion, FLIP is more promising as it gives detailed information about the whole length of the anal canal and as it is much easier to perform. FLIP provides detailed information about differences in diameter along the length of the anal canal, but a limiting factor could be that it is unable to detect variations along the circumference, because it estimates the anal canal as a circular cylinder. This will probably restrict the usefulness of FLIP in patients with lesions localized to part of the circumference including patients with traumatic or obstetric sphincter lesions. Pressure measurements as obtained with vector manometry do not have this limitation [18]. The spacing of the electrode pairs (5 mm) limits the spatial resolution of the FLIP. The rectoanal inhibitory reflex mediates relaxation of the IAS during rectal distension. This could be a potential source of error with FLIP as part of the bag is within the rectum. However, the diameters reached were much smaller than those previously needed to elicit the reflex [19], and a pilot test indicated that distension of the distal rectum to the small diameters within the range of the of the present study (35 mm) only lowered the anal pressure for a few seconds. The anal canal shortens during squeeze, but from the impression seen at the level of the puborectalis muscle during squeeze and by noting which electrodes were outside the anal verge during the experiment, we ensured that biomechanical properties were computed at the right levels. Movement of the probe within the anal canal during experiments should be considered a possible source of error. While the position was checked visually at each distension as described, movement between or at the beginning of distensions could theoretically cause contraction of the EAS.

The “hourglass” shape of the anal canal during distension with FLIP corresponded well with findings in a previous study using 3D ultrasonography [20]. Also, our finding that the mid-anal canal is the least distensible is in accordance with what we expected as this is the part where the IAS is thickest and also covered by the EAS [3]. The EAS is composed of fast and slow twitch muscle fibers, which allow it to maintain sustained tonic contraction at rest and also allow it to contract rapidly with voluntary squeeze [21]. Voluntary contraction of the EAS increases the pressure within the anal canal [4, 6], but in the range of CSA studied squeeze had surprisingly little effect on distensibility. An explanation for this could be that we only distended the anal canal to a diameter of a few millimeters. There are data showing that the contribution of the EAS to anal pressure increases with increasing diameters [22].

The functional role of the puborectalis muscle is difficult to evaluate [23]. Contraction of the muscle creates the anorectal angle which is important for continence and its relaxation during defecation allows the angle to become obtuse easing defecation [24]. Recent studies have indicated that the puborectalis muscle contributes to the anal pressure and closing mechanism [3, 20]. During squeeze, we clearly saw an impression corresponding to the muscle but still the upper part of the anal canal was the most distensible both at rest and during squeeze. Therefore, results from the present study indicate that the puborectalis muscle contributes to continence mainly by forming the anorectal angle and less by closing the anal canal. Accordingly, Liu et al. [3] showed that there was circumferential symmetry of anal canal pressures in the mid- and lower anal canal but not at the level of the puborectalis muscle.

The pathophysiology of fecal incontinence is multifactorial and varies between patient groups. There is no doubt that anal sphincter function is extremely important in maintaining continence and some authors state that most cases of fecal incontinence are due to defects and poor function of the EAS [25–27]. The clinical presentation of incontinence may represent different forms of sphincter dysfunction. Read et al. [25] found that incontinence to liquid stools mainly reflected low anal resting pressure while incontinence to solid stools reflected combined low anal resting and squeeze pressures. It is, however, likely that most cases of idiopathic fecal incontinence are multifactorial [28, 29], and future studies will reveal whether dynamic segmental evaluation with FLIP will contribute to the understanding of the pathophysiology of fecal incontinence. Furthermore, the authors find that biomechanical properties evaluated with FLIP may add new information about physiological changes in various other patient groups including patients with ileoanal pouch dysfunction or symptoms after anorectal irradiation therapy.

## Conclusions

FLIP has been modified to allow detailed information to be gathered about dynamic biomechanical properties along the length of the anal canal during distension. We have shown that these are nonuniform in accordance with the different structures surrounding the anal canal.

## References

[1] Jorge JM, Wexner SD (1993). Etiology and management of fecal incontinence. Dis Colon Rectum.

[2] Regadas F, Murad-Regadas S, Lima D (2007). Anal canal anatomy showed by three-dimensional anorectal ultrasonography. Surg Endosc.

[3] Liu J, Guaderrama N, Nager CW, Pretorius DH, Master S, Mittal RK (2006). Functional correlates of anal canal anatomy: puborectalis muscle and anal canal pressure. Am J Gastroenterol.

[4] Lestar B, Penninckx F, Kerremans R (1989). The composition of anal basal pressure. An in vivo and in vitro study in man. Int J Colorectal Dis.

[5] Bajwa A, Emmanuel A (2009). The physiology of continence and evacuation. Best Pract Res Clin Gastroenterol.

[6] Ryhammer AM, Laurberg S, Hermann AP (1997). Test-retest repeatability of anorectal physiology tests in healthy volunteers. Dis Colon Rectum.

[7] Santoro GA, Di Falco G, Santoro GA, Wieczorek P, Bartram CI (2010). Endoanal and endorectal ultrasonography. Pelvic floor disorders: imaging and multidisciplinary approach to management.

[8] Rasmussen OO, Colstrup H, Lose G, Christiansen J (1990). A technique for the dynamic assessment of anal sphincter function. Int J Colorectal Dis.

[9] Kwiatek MA, Pandolfino JE, Hirano I, Kahrilas PJ (2010). Esophagogastric junction distensibility assessed with an endoscopic functional luminal imaging probe (EndoFLIP). Gastrointest Endosc.

[10] McMahon BP, Frokjaer JB, Kunwald P (2007). The functional lumen imaging probe (FLIP) for evaluation of the esophagogastric junction. Am J Physiol Gastrointest Liver Physiol.

[11] McMahon BP, Frokjaer JB, Liao D, Kunwald P, Drewes AM, Gregersen H (2005). A new technique for evaluating sphincter function in visceral organs: application of the functional lumen imaging probe (FLIP) for the evaluation of the oesophago-gastric junction. Physiol Meas.

[12] Gregersen H, Orvar K, Christensen J (1992). Biomechanical properties of duodenal wall and duodenal tone during phase I and phase II of the MMC. Am J Physiol.

[13] Gregersen H, Stodkilde-Jorgensen H, Djurhuus JC, Mortensen SO (1988). The four-electrode impedance technique: a method for investigation of compliance in luminal organs. Clin Phys Physiol Meas.

[14] Gregersen H, Djurhuus JC (1991). Impedance planimetry: a new approach to biomechanical intestinal wall properties. Dig Dis.

[15] Freys SM, Fuchs KH, Fein M, Heimbucher J, Sailer M, Thiede A (1998). Inter- and intraindividual reproducibility of anorectal manometry. Langenbecks Arch Surg.

[16] Krogh K, Ryhammer AM, Lundby L, Gregersen H, Laurberg TS (2001). Comparison of methods used for measurement of rectal compliance. Dis Colon Rectum.

[17] Rasmussen OO, Sorensen M, Tetzschner T, Christiansen J (1992). Dynamic anal manometry: physiological variations and pathophysiological findings in fecal incontinence. Gastroenterology.

[18] Grande M, Cadeddu F, Sileri P (2011). Anal vector volume analysis: an effective tool in the management of pelvic floor disorders. Tech Coloproctol.

[19] Krogh K, Mosdal C, Gregersen H, Laurberg S (2002). Rectal wall properties in patients with acute and chronic spinal cord lesions. Dis Colon Rectum.

[20] Jung SA, Pretorius DH, Weinstein M, Nager CW, Den-Boer D, Mittal RK (2008). Closure mechanism of the anal canal in women: assessed by three-dimensional ultrasound imaging. Dis Colon Rectum.

[21] Konsten J, Baeten CG, Havenith MG, Soeters PB (1993). Morphology of dynamic graciloplasty compared with the anal sphincter. Dis Colon Rectum.

[22] Duthie HL, Kwong NK, Brown B (1970). Adaptability of the anal canal to distension. Br J Surg.

[23] Azpiroz F, Enck P, Whitehead WE (2002). Anorectal functional testing: review of collective experience. Am J Gastroenterol.

[24] Fletcher JG, Busse RF, Riederer SJ (2003). Magnetic resonance imaging of anatomic and dynamic defects of the pelvic floor in defecatory disorders. Am J Gastroenterol.

[25] Read NW, Bartolo DC, Read MG (1984). Differences in anal function in patients with incontinence to solids and in patients with incontinence to liquids. Br J Surg.

[26] Sultan AH, Kamm MA, Hudson CN, Thomas JM, Bartram CI (1993). Anal-sphincter disruption during vaginal delivery. N Engl J Med.

[27] Read NW, Haynes WG, Bartolo DC (1983). Use of anorectal manometry during rectal infusion of saline to investigate sphincter function in incontinent patients. Gastroenterology.

[28] Bharucha AE, Fletcher JG, Harper CM (2005). Relationship between symptoms and disordered continence mechanisms in women with idiopathic faecal incontinence. Gut.

[29] Fernandez-Fraga X, Azpiroz F, Malagelada JR (2002). Significance of pelvic floor muscles in anal incontinence. Gastroenterology.

